# Case of resolution of plaque psoriasis following treatment with rifampicin for latent tuberculosis: A case report

**DOI:** 10.1177/2050313X231152054

**Published:** 2023-02-06

**Authors:** Anastasiya Muntyanu, Sylvia A Martinez-Cabriales, Jensen Yeung

**Affiliations:** 1 Division of Dermatology, Department of Medicine, University of Toronto, Toronto, ON, Canada; 2 Division of Clinical Sciences, Department of Medicine, Northern Ontario School of Medicine, Sudbury, ON, Canada; 3 School of Medicine, Autonomous University of Nuevo Leon, Monterrey, Mexico; 4 Division of Dermatology, Women’s College Hospital, Toronto, ON, Canada

**Keywords:** Psoriasis, tuberculosis, rifampicin, biologics, infection

## Abstract

Psoriasis is a common cutaneous disease, and often these patients require treatment with biologics. Screening for latent tuberculosis is an important step in the pre-biologic work-up. A 58-year-old woman with moderate-to-severe psoriasis was found to be positive for latent tuberculosis during pre-biologic screening. She received rifampicin for 6 months and had complete resolution of her psoriasis, with persistent remission at 1-year follow-up. Improvement could be attributed to the immunomodulatory effects of rifampicin. Another theory proposes the existence of a tuberculosis-related type of psoriasis that improves when tuberculosis is adequately treated.

## Introduction

Screening for latent tuberculosis (LTB) is an important step in the pre-biologic work-up for patients starting biologics for psoriasis. If the patient is found to be positive for LTB, first-line treatment includes daily rifampicin for 4 months^1^ or isoniazid daily for 9 months. Patients can start a biologic after 1 month of LTB treatment. The prevalence of LTB in patients with psoriasis who are candidates for systemic therapy ranges from 4% to 14%.^2,3^ Here, we report a case of a patient with moderate-to-severe plaque psoriasis who resolved with rifampicin treatment of LTB.

## Case report

A 58-year-old woman, originally from Somalia, presented to our Dermatology clinic for assessment of a relapsing course of generalized pruritic eruption. Her comorbidities included type 2 diabetes, hypertension, dyslipidemia, non-alcoholic fatty liver disease, peripheral artery disease, and immune thrombocytopenic purpura. She reported that her skin lesions improved with sun exposure and worsened in the winter. Previous treatments included only calcipotriol and betamethasone dipropionate ointment to which she had minimal improvement. On physical examination, there were widespread scaly erythematous papules and plaques, affecting 10% of her body surface area (BSA). Halobetasol propionate cream was started while awaiting histopathology results that finally confirmed a diagnosis of psoriasis.

As a treatment for her severe psoriasis (baseline BSA 10%, Psoriasis Area Severity Index (PASI) of 10.2; Dermatology Life Quality Index (DLQI) of 15) and patient preference against a biologic therapy at this time, apremilast 30 mg twice daily was initiated. After 16 weeks of treatment, her psoriasis significantly improved (BSA of 2%, PASI of 2.6). However, after 4 months of treatment, the dosing had to be reduced to 30 mg daily due to gastrointestinal side effects. Following this dose modification, her psoriasis worsened (BSA of 15%; PASI of 12.8), and to prepare her for the next therapeutic option, we performed a pre-immunosuppressive work-up that included blood test, TB skin test, and chest X-ray (CXR).

At this pre-biologic screening, she was diagnosed with LTB by the infectious diseases team. She had an abnormal CXR, and positive TB skin test and QuantiFERON-TB Gold without any symptoms of active TB. A computed tomography (CT) chest scan was ordered for further assessment, showing a small region of chronic inflammatory reaction in the posterior area of the left upper lobe and a superimposed granuloma. She was treated with rifampicin 600 mg daily for 4 months, and much to our surprise, her psoriasis completely resolved within 2–3 months of treatment. In addition, her psoriasis has remained clear 1 year after stopping rifampicin.

## Discussion

Over the last few decades, several studies in the literature have assessed the use of rifampicin in the treatment of psoriasis. The benefits observed were thought to be attributed to its immunomodulatory effects.^4^ A later study by Gupta et al.^5^ found significant suppression of T lymphocytes 2–3 weeks after rifampicin therapy. Another study found that rifampicin reduced interleukin (IL)-1β and tumor necrosis factor (TNF)-α production while increased anti-inflammatory cytokine IL-10.^4^

In addition, one case report described a patient with a 15-year history of psoriasis, who experienced significant improvement of cutaneous lesions within 1 month of treatment with rifampicin for active pulmonary TB. However, he interrupted therapy which led to a flare of his psoriasis. But he once again responded when rifampicin therapy was reinstated.^6^ In a clinical trial of 25 patients with chronic plaque psoriasis, those treated with rifampicin showed a marked improvement, as PASI 75 was shown in 3 patients while PASI 50 was seen in 13 patients after 60 days of treatment.^2^ The immunomodulatory, rather than antimicrobial, effects of rifampicin in eruptive psoriasis were supported by studies showing a significant PASI reduction in both patients with concomitant streptococcal infection and those without. Specifically, of the 82 patients with chronic plaque psoriasis (39 with evidence of streptococcal infection and 43 without), PASI 75 was achieved in 44%–55% of patients treated with rifampicin 600 mg daily for 60 days,^4^ and no difference was detected between the group with evidence of infection and the group without.

Another theory proposes the existence of a tuberculosis-related type of psoriasis that improves when tuberculosis is adequately treated.^2^ This is similar to guttate psoriasis, which is frequently associated with a streptococcal infection, and when treated could substantially lead to improvement of psoriasis. This was supported by multiple case reports showing clearance of psoriasis after treatment of LTB with isoniazid.^7^ However, it is important to consider the possible immunomodulatory effects of isoniazid, but this has not been clearly elucidated.

For our patient, the long-standing plaque psoriasis completely cleared after 3 months of rifampicin therapy. At the 1-year follow-up, she remained in remission with no recurrence of psoriasis lesions.

Rifampicin has also shown efficacy both in in vivo and in vitro studies in several other inflammatory cutaneous diseases including atopic dermatitis (AD) and hidradenitis suppurativa (HS). In AD, rifampicin led to decreased release of inflammatory mediators, TNF-α and prostaglandin D2 in mast cells as well as a concentration-dependent reduction of mRNA expression of cyclooxygenase 2.^8^ For HS, anti-inflammatory effect was attributed to decreased production of IL-1β, IL-6, IL-8, IL-10, and TNF-α.^9^ Hence, rifampicin may be an effective therapy in some patients with inflammatory skin diseases due to its immunomodulatory effects.
